# Navigating life after multiple amputations: a qualitative exploration of rehabilitation and everyday challenges in Norway

**DOI:** 10.3389/fresc.2025.1542441

**Published:** 2025-08-07

**Authors:** Nina Enersen, Daniel Løke, Kirsti Skavberg Roaldsen, Randi Sviland

**Affiliations:** ^1^ Department for Multitrauma, Neurology and Burns, Sunnaas Rehabilitation Hospital, Bjørnemyr, Norway; ^2^ Department for Functional Assessment, Sunnaas Rehabilitation Hospital, Bjørnemyr, Norway; ^3^ Child and Youth Clinic, Department of Habilitation, Akershus University Hospital, Lørenskog, Norway; ^4^ Department for Research, Sunnaas Rehabilitation Hospital, Bjørnemyr, Norway; ^5^ Department of Neurobiology, Care Sciences and Society, Karolinska Institutet, Stockholm, Sweden; ^6^ Faculty of Health Sciences, Department of Health and Care Sciences, UiT The Arctic University of Norway, Tromsø, Norway; ^7^ Faculty of Health and Social Sciences, Department of Health and Functioning, Western Norway University of Applied Sciences, Bergen, Norway

**Keywords:** multiple amputations, limb loss, lifeworld, rehabilitation, disability

## Abstract

**Purpose:**

Multiple amputations are a rare outcome following critical illness or injury, with significant impacts on the lives of those affected. This study aimed to explore and describe experiences of everyday life and municipal rehabilitation services among individuals with multiple amputations after their discharge from specialized rehabilitation in Norway.

**Methods:**

A qualitative research design was used with a lifeworld phenomenology perspective. Data were collected through individual, semi-structured interviews with five community-dwelling adults—one man and four women—who had multiple amputations, including at least one upper extremity. Data were analyzed using systematic text condensation.

**Result:**

Four categories emerged to describe the challenges of living with multiple amputations: “Navigating Dependence and Bodily Limitations”, “Challenges in Regaining Autonomy”, “Rehabilitation Challenges and Adjusting Expectations”, and “Adapting to a New Normal”.

**Conclusion:**

Participants experienced dependence, vulnerability, and a restricted lifeworld. The contrast between life with and without prosthetics underscores their vital role in autonomy. Unmet expectations of local rehabilitation services reveal structural barriers. The study highlights their experiences, advocating a lifeworld perspective to improve rehabilitation services.

## 1 Introduction

Multiple amputations affecting upper and lower extremities are a rare but devastating consequence of severe trauma or illness, most often resulting from war injuries, high-voltage accidents, burns, or symmetric peripheral gangrene following sepsis (1). Prevalence typically varies based on the cause of amputation. In the United States, multiple amputations represent only 7.3% of all traumatic amputations, excluding those related to war (2). A retrospective analysis of amputations in the U.S. Military from 2001 to 2010 showed an average amputation rate of 5.3 per 100,000 deployed troops, with 30% of all amputations involving multiple limbs (3). In Europe, the number of multiple amputation cases is expected to rise due to the ongoing war in Ukraine. In countries like Norway, where labor safety regulations, road safety measures, and the absence of armed conflict are well-established, the prevalence of multiple amputations is presumed to be low. However, no current data exists to confirm this. Sepsis, a life-threatening condition affecting approximately 50 million people globally each year (4), can also lead to amputations. One-sixth of survivors experience severe long-term impairment, including functional limitations, cognitive deficits, and mental health challenges (5). Amputations occur in about two out of every 1,000 patients with vasopressor-dependent sepsis (6).

Regardless of the cause, multiple amputations have a profound impact on an individual's life. Survivors require comprehensive rehabilitation services, which are typically provided in specialized rehabilitation facilities. Following discharge, continued follow-up is necessary within the community (7). In comparison to the more common unilateral leg amputations, often caused by peripheral vascular disease or diabetes, individuals with multiple amputations tend to be younger and have fewer pre-existing comorbidities prior to the amputation (2). As a result, they may possess greater rehabilitation potential (8). However, survivors of critical illness or trauma frequently experience additional injuries or long-term complications alongside the sudden and drastic changes to their lives. These factors can significantly influence their psychological well-being (7).

It is documented that amputations can affect a broad range of physical, psychological, and social functioning (9–12). People with amputations suffer from more pain interference, worse physical function and dissatisfaction with social roles than the norm (10). And further, have more anxiety, depression and body image disorders, and prosthetic use may influence psychosocial adjustment (11). Studies also suggest that upper-limb amputations have a greater impact on body image, anxiety, and social discomfort (13). Individuals with lower-limb amputation might experience a restricted lifeworld due to decreased physical and social mobility (14). Research also indicates that persons with multiple amputations after sepsis report lower health-related quality of life compared to the general population (15). Coping strategies for adapting to a new situation after a unilateral amputation include engaging in meaningful activities (16, 17) and seeking professional, social, and emotional support from friends and family (17–20). However, the literature is primarily focused on the impact of losing one limb, and life experiences, barriers to social participation and coping mechanisms for people experiencing multiple limb loss is less explored.

The aim of the study was to explore how individuals with multiple amputations experience reintegration into daily life following specialized rehabilitation and how their altered bodies impact their lives. Additionally, the study sought to provide insights that could enhance community rehabilitation services for this population. By exploring their lived experiences, clinicians, service providers, policymakers, and researchers can gain valuable knowledge to improve rehabilitation support and ensure more effective community-based care.

## 2 Theoretical perspective

Phenomenology explores human experiences by focusing on how individuals perceive and make meaning of their lives. Husserl's phenomenological lifeworld concept offers a perspective on how individuals experience being, acting and interacting in everyday life. The lifeworld is the backdrop of our lives, shaped by experiences and meanings that are taken for granted and remain implicit (21). Meaning is shaped through experience. Merleau-Ponty expands on this by highlighting the body's role in perception, arguing that our lived experiences are always mediated with our bodily existence through our senses, perceptions and emotions (22). As embodied beings, our interactions with the environment, others, and ourselves shape how we understand and experience the world (23). From this perspective, physical differences, such as disability, influence the individuals perception of everyday life, highlighting the existential dimension of embodiment (22). The body is the fundamental starting point from which all human experiences unfold (22).

In this study, we employed an inductive approach. Our systematic interpretation of the material highlighted lived experiences of returning to life after multiple amputations and a phenomenological lifeworld perspective emerged as the most relevant vantage point for discussing and enlightening our findings.

## 3 Context

In Norway, there is universal health care in both specialist and primary health care services primarily funded through public sources ensuring access to all regardless of income (24). Complex rehabilitation, such as that required for individuals with multiple amputations, is initially managed by specialist hospitals operated by governmental regional health authorities. Following discharge, responsibility for rehabilitation, home care services, and assistive technology shifts to the community rehabilitation system. In 2012 a Coordination Reform transferred more responsibility for rehabilitation services and long-term follow-up to municipalities. This shift has led to reports of patient groups, including individuals with amputations, facing challenges with discontinuity of care and increasingly fragmented services (25).

The United Kingdom provides a similar model of publicly funded health care through the National Health Service (NHS) which emphasize long term-support and community-based rehabilitation, however the rehabilitation services are fragmented with regional variations (26). In the United States, the healthcare system is largely private and dependent on insurance coverage (26), which may lead to differences in quality and affordability of services.

## 4 Method

In this study, we wanted to explore and describe the experiences of living with multiple amputations and applied a qualitative research approach using semi-structured interviews. This approach was chosen to provide an in-depth understanding of the complexity of this disability by examining the unique experiences of specific individuals (27). The COREQ guidelines were adhered to for reporting the study's results (28). Ethical approval was obtained from the Norwegian Centre for Research Data (809713), and the Regional Ethics Committee deemed the project exempt from formal review (350836). All participants signed a written consent form prior to the study, which outlined the aim of the study, their right to withdraw, and the confidentiality of their data.

### 4.1 Participants

Convenience sampling was employed through a user organization and a rehabilitation institution. These organizations used their networks to reach out to potential participants, who could contact the research team if interested in receiving both verbal and written information about the study. Participants were eligible to join the study if they had two or more major amputations, including at least one hand, and had been discharged home from specialized rehabilitation more than six months prior. Individuals were excluded if they had concurrent medical conditions that overshadowed the experience of living with multiple amputations, limited proficiency in Norwegian, or only lower-limb amputations.

Five individuals with multiple amputations made contact, and all five consented to participate in the study. The group consisted of one man and four women, with a median age of 53 years (range: 33–77). Three participants had major amputations in all four extremities, while two had three major amputations and one minor amputation (e.g., partial foot or hand amputation). The primary cause of amputation was symmetrical peripheral gangrene. It had been a median of eight years (range: 3–17) since the amputations, and the median age at the time of amputation was 46 years (range: 16–74).

### 4.2 Data collection

The interviews and transcriptions were conducted by the first author (NE), an experienced physiotherapist in specialized rehabilitation for persons with multiple amputations. The co-authors are researchers and health professionals with backgrounds in physiotherapy and psychology.

Individual interviews were conducted in February 2022 at the participants’ homes, according to their preference. A semi-structured interview guide was used, (see Table 1). The interview guide was pilot tested on a peer mentor with a cervical spinal cord injury and experience in qualitative research, leading to minor revisions. The interviews were conducted during the later stages of the COVID 19 pandemic, but the aim was not to explore the impact of the pandemic on health care services. The participants were asked to recall the transition to community services, which for all was before the pandemic started. The first author took notes on immediate thoughts and reflections during the interviews, which were included in the analysis of the data. The interviews lasted an average of 60 min and were audio-recorded, with subsequent verbatim transcription.

**Table 1:** Interview guide with guiding questions and themes.

Main question	Guiding questions/themes
I would like us to start by you telling me a little about what it is like for you to live with multiple amputations.	How does it affect relationships? - Family life as a mother, father, grandparent, or partner - Social life with friends
	How does it affect interaction with your surroundings or mobility? - Work, voluntary positions, or meaningful activities - Assistive devices, technology, or car
	What limits or enables you to live the life you want to live or do the things you want to do?
	Can you describe the most positive or negative thing that has happened to you as a result of the amputations?
Now, let's change the topic. I would like you to tell me a little about what it was like to come home again (after rehabilitation).	What were your thoughts about coming home? (worries or expectations)
	How did you “find your way back to everyday life”?
	If you were to do it again, is there anything you would do differently?
	What do you wish you had known?
Lastly, what advice would you give to:	- Persons with newly acquired multiple amputations? - Those working in the municipal support system or rehabilitation services?
Do you think you've said what's important to you about this topic?	Is there anything you would like to add that we haven't discussed?

### 4.3 Data analysis and rigor

Data were analyzed using systematic text condensation (29), a dynamic four-step cross-case analysis process. The analysis began with iterative readings of the transcriptions and discussions between the first author (NE) and the last author (RS), which led to the identification of four preliminary themes. Based on these themes, meaningful units were coded into groups with subcodes. NVivo software was used to systematize the data (30). Meaningful units in the subgroups were then condensed into a first-person perspective, and an illustrative “golden quote” was selected. Finally, the condensate was transformed into analytical text and presented in result categories (29). Rather than following a linear process, the analysis was iterative, with repeated back-and-forth steps to cross-check codes, subcodes, and condensates. Each step of the analysis was presented to the co-authors and thoroughly discussed to reach consensus. The analytical steps were mainly conducted by one researcher, with potential limitations in perspective and bias mitigated through discussions with the research team for validation and reflexivity. The research team engaged in reflective discussions to challenge potential preconceived attitudes.

## 5 Results

The data analysis resulted in four result categories that describe experiences with everyday life and rehabilitation services after specialized rehabilitation: “Navigating Dependence and Bodily Limitations”, “Challenges in Regaining Autonomy”, “Rehabilitation Challenges and Adjusting Expectations”, and “Adapting to a New Normal”. Quotes from the interviews, with gender-neutral names for anonymization, are used to illustrate the categories.

### 5.1 Navigating dependence and bodily limitations

Participants describe an embodied sense of entrapment, as if their disabled bodies have become an inescapable cage without the prosthetics on. They recount moments of helplessness and vulnerability in situations they cannot escape or control. This vulnerability is also expressed a tendency to avoidance of situations where they must depend on others, as becoming dependent on others is a difficult adjustment for the participants. They feel uncomfortable with the visibility of their disability and are hesitant to show their bodies to others.

“So there I was, lying in bed without my prosthetics—and in that moment, you are trapped. That feeling of being trapped, unable to get out of bed, unable to do anything except call for help, still lingers from my time in the hospital. And it’s tough, really tough,” (Jules).

All participants describe a profound loss of self-reliance and freedom - an erosion that transforms the everyday into a constant negotiation with dependence. They portrait freedom as the intrinsic capability to manage even the most basic daily tasks, such as getting out of bed, using the restroom, getting dressed, and eating. Some participants must retire to bed before their spouse each night because they need assistance, while others find themselves sitting in their pajamas well into the late morning, waiting for help to get dressed—losing an unacknowledged freedom in their lifeworld. The participants describe the ability to don or remove prosthetics, and to access necessary aids and environmental adjustments as necessary in reclaiming that sense of self-reliance and autonomy.

“I am completely dependent on others to get up in the morning and put on my prosthetics. It’s a hassle, so I’m working on figuring it out. Now, I can go to bed by myself—as long as I go before him [the spouse]—because he has to tidy up my clothes, prepare my prosthetics, wash the liners, and set them to charge*,*” (Alex).

The participants express an acute sense of being marginalized in their own lives. Their new bodies impose limitations on engaging with their grandchildren, participating in physical and social activities, or even simply stepping outside. Lack of accessibility in the physical environment, dependence on specialized toilets, or reliance on assistants, becomes a constant reminder of their altered lifeworld. Several participants explain that they can still participate in certain activities, but it requires extensive planning and time investment, stripping away much of the spontaneous joy that once derived from those activities.

“It becomes very painful. I can’t stroke her cheek, pull up a zipper, or put on a jacket—all those practical things. It’s quite painful when you can’t participate at the play center, other than just sitting and watching. Before this happened, I used to join in, jumping on trampolines and crawling around. I can’t do that anymore. You get a completely different life,” (Kim).

### 5.2 Challenges in regaining autonomy

Participants describe the intrusion of home care services into their personal spaces as deeply invasive, with “*all kinds of people*” entering their homes despite the necessity of their support. It is especially difficult to receive assistance with the most intimate tasks. They struggle to maintain control over their immediate lifeworld, but are at the mercy of the personal preferences and time constraints of the home care services. Some have chosen personal assistants to reclaim a degree of autonomy, while others, preferring solitude, reluctantly accept the unpredictable rhythm of the home care service as part of their altered everyday existence.

“Regarding home care services: “It certainly felt like an invasion. I'm not used to anything like that. But you just have to accept that this is how it is now. If you need help, people have to come in,” (Luca).

Participants receiving home care services describe the ongoing investment of time and energy required to train their helpers, a process that becomes an added burden in their already demanding lives. Each new caregiver necessitates a detailed explanation of their specific needs and preferences, particularly regarding the intimate routines within their homes. While they recognize the necessity of this guidance for effective support, there are moments when they feel depleted—lacking the energy and patience to continuously navigate this training process, which further intensifies their sense of dependence and vulnerability.

“I get irritated. I use so much energy. When you’re an amputee like me, you get tired more quickly, right? So I spend a lot of energy just explaining everything—like in the shower, telling them where the shampoo is, what to do next, how to do it, which towel to use—but not that one. I’m talking nonstop during those moments, and I just want it to be efficient.*”* (Jules).

The participants describe their new lives as profoundly strenuous, with each day requires extensive planning. Whether it is managing the hours allocated for personal assistance, navigating the complexities of healthcare appointments and unpredictable patient transportation, or simply planning an outing, every task requires careful coordination. They need to familiarize themselves with the accessibility of their destinations, allow enough time for transport and mobility, and coordinate their activities with any assisting personnel. To maintain a sense of control, this requires the ability to always stay ahead of the situations, which continuously drains their energy.

“I think you have to experience it yourself or have it so close within your own family to truly understand what this is like. Because it’s so difficult. You have to be on top of everything all the time. And of course, I get exhausted because there’s so much I have to manage. I always have to stay ahead—schedules, booking taxis, being ready. Then the taxis don’t show up, so I have to follow up, and then it’s a whole ordeal. There are just so many little things that are so draining.*”* (Luca).

### 5.3 Rehabilitation challenges and adjusting expectations

The participants share stories of shattered dreams related to their encounter with the rehabilitation services in their communities. They describe how a lack of essential services, equipment, expertise, and continuity undermines the sense of progress they initially experienced during rehabilitation. Those with access to physiotherapists describe short and ineffective sessions, such as fifteen minutes of prosthetic training per week. The participants express a need for continuous support over several years to achieve a higher level of independence. Most of them desire more rehabilitation and tailored training to maintain function and health, but one participant lacks motivation for this.

“There have been many obstacles since I came home. When I left rehabilitation, I thought, “In a year, I’ll be able to walk through the door on my own.” I had such high expectations and believed anything was possible—then everything just came crashing down like a house of cards,” (Kim).

Frustration and a sense of powerlessness arise when dealing with the community rehabilitation services, according to the participants. One of them mentions feeling as though they have no rights. They describe lengthy processes and waiting for weeks to receive help when their wheelchair's battery is broken, or the automatic door fails. They also share experiences of lack of involvement and understanding from the service providers. Several participants express resignation and carefully choose their battles.

“Every time there’s something wrong, it’s the same lesson. If there’s an issue with that pump [automatic door], I can’t just call NAV [Norwegian Labour and Welfare Administration] and tell them they need to come help me and fix it. No, I have to call the municipality, and then they have to call NAV to ask if they can do something about it. Then NAV has to call and order the person who’s supposed to fix it. I have the number to the guy—I could just call him myself—but it’s not allowed. I have to go through the municipality to get it done,” (Luca).

The participants express expectations for healthcare professionals to possess expertise in the field of amputations. One participant recounted receiving a new hand prosthesis without prior consultation about size and shape, finding it perplexing that orthopedic engineers failed to recognize the importance of user input. Several individuals have encountered physiotherapists who cannot provide rehabilitation tailored to their specific needs. Furthermore, some individuals have been assigned intern physiotherapists who lack expertise in prosthetic training, leading to the exclusion of this training despite the participants’ expressed desire for it.

“They were surprised that I already had prosthetics—but I had been using them for over six months. They hadn’t even realized. So I asked, “What are we going to train on here?” and they said they couldn’t offer anything more than what I had already received at [specialized rehab center], so there was nothing specific to train on. That was really depressing (…) I had expected to get training in using my hands, but the staff there were completely useless,” (Alex).

### 5.4 Adapting to a new normal

The participants all emphasize the pivotal role of rehabilitation in shaping their current lives. They recount relying heavily on health care professionals for relearning essential skills, which in turn fostered a growing sense of self-confidence and belief in their own abilities. Home-based rehabilitation was important in finding solutions to everyday challenges that arise upon returning home. They also stress the importance of healthcare professionals who accompany them closely throughout their rehabilitation journey, gaining a deep understanding of their unique needs and providing tailored support.

“ Fortunately, they [community rehabilitation team] came to my home. I didn't have them for long, but it was very important. The occupational therapist observed how I did things and offered simple suggestions. In the beginning, you don't come up with solutions on your own, you don't stand a chance” (Kim).

Meaningful activities are described as crucial for the process of rediscovering themself. As part of managing chaotic thoughts and finding inner peace, several participants have engaged in creative hobbies during the rehabilitation process and in their everyday lives thereafter. For some, having assistive devices, like powered wheelchairs, to access nature and engage in physical activities is essential. One participant describes returning to the ski slopes as reclaiming a part of herself. However, rediscovering oneself within the confines of a changed body is described as a complex and ongoing challenge. Many believe it is significant to utilize their skills and be of use to others, as it allows them to experience a sense of fulfillment and allows their functional limitations recede into the background.

“I love skiing, and I actually started again two weeks ago. It’s fun, but I just can't do it the way I used to. It feels like I've rediscovered a part of myself, yet it’s not quite the same. It opens up a new door that had been closed. Before the accident, I was primarily a skier. So getting back on the slopes was like finding a piece of myself again,” (Taylor).

Close friends and colleagues emerge as vital sources of support for several participants, providing them with strength and encouragement throughout their journey. One participant recount how her friends played a pivotal role in getting her advanced prosthesis, enabling her the ability to walk after several years in a wheelchair, which brought a new dimension to her life. Another participant recalls how her employer insisted on her returning to work. In contrast, another participant shares the absence of support from her employer, which ultimately caused her to abandon the idea of returning to work. These contrasting experiences illustrate how the presence or absence of support can profoundly shape the participants’ sense of self and future possibilities.

“Also, I had complete freedom at the start—I could come and go as I pleased, just needing to figure out what accommodations I required. (…) Having an employer who believes in you is crucial. If you feel your employer doesn't believe in you, it’s hard to motivate yourself. In the beginning, you need external motivation because you don't yet have enough within yourself. You just don’t,” (Jules).

The participants describe a gradual process of acceptance of their altered reality. Despite the increased demands that come with their new lives, they have adapted to this new normal, which has become integral to their sense of being in the world. Even many years after their amputations, they are experiencing continuous progress in their functioning. Prosthetics have become an increasingly integrated part of their identity, with prosthetic technology offering new possibilities. They constantly discover new and creative solutions to everyday challenges. One participant describes using prosthetics as an art form, but once you master it, you can have a much better life, where the challenges that once seemed overwhelming and difficult no longer hold the same weight.

“It is possible to have a decent life even after losing limbs. I lost four parts of myself, but the rest of me still works” (Kim).

## 6 Discussion

Our study explored the lived experiences of individuals with multiple amputations after discharge from specialized rehabilitation. Although the number of participants is limited, the result categories reveal a compelling picture of their reality: being-in-the-world in a dependent and vulnerable body that constrains their lifeworld, daily struggles in managing barriers in the environment, and ordeals experienced when engaging with rehabilitation services. The study also highlights the importance of professional and social support, as well as the pursuit of meaningful activities, in their journey to reclaim their lives.

### 6.1 The struggle for autonomy in a transformed body

The study highlights the profound challenges of adapting to a dramatically altered body after sudden trauma or illness, marked by substantial loss of physical function that abruptly alters their lifeworld.

Firstly, the participants describe a sense of being trapped in their bodies, facing a multitude of impossibilities in daily life. This sense of entrapment coupled with a dependence on others, exposes vulnerability and weakness. These findings align with research on upper limb amputations (2, 16) and lower limb amputations (14, 31, 32). However, a key distinction emerges in how the participants experience life with and without prosthetics. Without prosthetics, they are nearly incapable of performing any task, thus severely limiting their lifeworld to their bed or wherever they are confined. The need for caregivers becomes essential during these moments. In contrast, when prosthetics are worn, they enable a different functionality, allowing participants to maintain a certain degree of independence. Prosthetics enable them to engage with the world, and their participation depends heavily on these devices. When the prosthetics are removed—whether during the night or in activities such as bathing—that the true impact of their disability comes into sharp focus. In these moments, participants describe an overwhelming sense of entrapment. While prosthetics offer a sense of autonomy and capability, the reliance on others during moments without them challenges the participants’ previous sense of self, threatening their identity as autonomous individuals (33). Toombs has described, through her personal experience living with multiple sclerosis, how injury or disease can turn the body into an object beyond one's control, thus disrupting a person's lifeworld (23). Being-in-the-world then becomes a state of being a body while simultaneously feeling separated or alienated from that body. As the bodies of our participants become the source of loss of autonomy and a hindrance to life fulfilment, it creates a stark contrast to the integrated unity of self and body. Other studies have described a shrinking lifeworld due to loss of freedom and independence following lower limb amputations (14). Our study portrays experiences of their new bodies limiting their lifeworld to the periphery of existence.

Secondly, the participants portray a highly demanding life. The participants encounter difficulties beyond the limitations of mobility and self-sufficiency, highlighting additional barriers such as inaccessible environments, transportation difficulties, and a community rehabilitation system that falls short of meeting their needs. Everyday activities that were once easily attainable have become impossible, or immensely time consuming. Some individuals find themselves isolated in their homes with no clear strategy to overcome the environmental barriers, further diminishing their lifeworld and threatening their social existence. Other researchers have highlighted the cognitive burden associated with mobility after amputation due to increased attentional demands for physical tasks (17, 34, 35). Additionally, individuals with physical impairments often face a heightened need for deliberate planning and consideration in daily activities (32, 36). These findings align with our study, but the challenges of living with multiple amputations may be even greater than those experienced with a single amputation. In addition to the increased mental and physical effort required for walking and doing daily tasks with multiple prosthetics, the participants describe becoming fatigued by the need to constantly train the caregivers to meet their specific needs, and expressed disillusionment with the constant need to advocate for themselves, repeatedly justifying and negotiating for essential services to support their daily functioning. This persistent struggle added an emotional and psychological burden, further complicating their adjustment to life after amputation. Navigating the complexities of the service systems and interacting with their personnel leaves the participants feeling drained. This resonates with Toombs’ notion of the encounter with an obstructive and unaccommodating world so exhausting that it leads to a state of existential fatigue (23). When every demand in their everyday life feels like an existential threat, we should ask how can the service system lessen the burden.

### 6.2 Between hope and reality: navigating rehabilitation services

The new limitations on their physical abilities create a profound sense of impossibility in daily life, posing a threat to the participants’ sense of identity and existential integrity. A transformative journey unfolds during the initial rehabilitation. At discharge, a palpable hope for the future was imminent, but the participants encountered gaps between their expectations and the reality of available services.

In Norway, where this study was conducted, the social welfare system is recognized for its comprehensive support, offering universal access to healthcare, community rehabilitation services, and the right to essential assistive devices at no cost (37). This might also set high expectations, as participants expressed dissatisfaction with the service system. Their frustrations stem from long processes, challenges in obtaining necessary aids and physiotherapy services, and the sense that they constantly have to “fight for their rights”. As previously discussed, living with a disability can be exhausting, and this burden can be worsened by a complex, bureaucratic system that complicates access to essential services, making navigation a struggle. Structural issues in physiotherapy provision also contributed to dissatisfaction with the rehabilitation services. Physiotherapists in primary health care may be employed by the municipality, have contractual agreements with the municipality, or operate privately, and contracted physiotherapists have no financial incentives to prioritize individuals with severe disabilities (38). Additionally, the lack of universally accessible rehabilitation facilities limits opportunities for this population beyond specialized care (39).

Since 2012, Norway has undergone a decentralization of rehabilitation services, which has led to documented shortcomings (40). In a small country with many rural communities, it is also challenging to maintain specialized knowledge and expertise across all regions. This raises the question of whether certain aspects of rehabilitation should remain centralized to ensure consistent, high-quality care. Given the complexity of multiple prosthetics, we argue for lifelong follow up services, ideally within established amputation clinics and rehabilitation centers that specialize in amputations. Effective, interdisciplinary rehabilitation services offer not only practical support but also a sense of security and belonging, addressing the existential challenges faced by individuals navigating life after amputation.

Home care services are, by nature, decentralized, and with an aging population, the strain on these services is only increasing. While not a new argument, investing in rehabilitation that promotes independence could benefit both patients and reduce the long-term burden on home care services. By enabling individuals to regain functional autonomy, the demand for long-term assistance may alleviated, ultimately leading individual and socio-economic benefits (41).

### 6.3 Rehabilitation as a path to repossibility

Our study highlights that rehabilitation for multiple amputees requires broader insights. The stark contrast between feeling trapped in one's body without prosthetics and regaining some function with them underscores the importance of intensive training in certain areas. Beyond walking, individuals need functional training in being independent without prosthetics, learning to manage donning and doffing, as well as managing the practical aspects of prosthetic use in everyday use such as cleaning, charging and preparing for the next day. Research indicates that individuals with upper limb amputations do not always prioritize using arm prosthetics in daily activities, and that sufficient training is paramount (42). For multiple amputees, mastering prosthetic use early in rehabilitation is even more crucial, as it would likely expands their lifeworld and increases their independence.

Rehabilitation aims to restore or improve function and quality of life from a biopsychosocial perspective (43). The phenomenological lifeworld perspective emphasizes the deeply personal and subjective impact of disability, recognizing that the challenges and impossibilities experienced are unique to the individual (44). For one person, the loss of mobility may mean giving up skiing, while for another, skiing may never have been relevant in the first place. Rehabilitation, then, is about reclaiming a meaningful life in new ways. Our study presents two compelling examples of this reclamation; one participant, with support from friends, was able to return to skiing with new prosthetics, reconnecting with a sense of self. Another found newfound freedom though an “off-piste” powered wheelchair, that expanded her lifeworld and feeling recognized as a person with unique needs. Comprehensive, care-oriented approaches are preferred by patients (17, 19), and healthcare professionals recognize the importance of understanding patients’ experiences and contexts (45).

This underscores that the pursuit of rebuilding a life after multiple amputations is not an individual endeavor but a deeply social construct involving rehabilitation workers, service providers, assistive technology, environment, social networks, and the individual themselves. From a phenomenological perspective, recovery can be described as “re-possibilizing the world” (44). This perspective highlights the social responsibility of healthcare and social services to create opportunities for individuals to lead meaningful lives based on their unique needs. Consistent with our results, engaging in meaningful activities (16, 17) and receiving professional and social support from friends and family (17–19) have been identified as crucial aspects of regaining a sense of normalcy and freedom. Therefore, community rehabilitation services should prioritize facilitating meaningful activities within a social context. In the pursuit of cost-effective and evidence-based approaches in rehabilitation, it is essential to not overlook the lived experiences of patients, and reducing them to mere objects in the system. A phenomenological approach to rehabilitation requires integrating the individual's previous social situation, lifeworld, and personal perspectives when designing interventions. Only by deeply understanding each person's experience can rehabilitation services truly unlock new possibilities and provide meaningful support for those adapting to life after multiple amputations.

## 7 Limitations

In qualitative research, we acknowledge that the choices we make, based on our experience and perspective, influence both data collection and analysis, necessitating a reflective practice. The study has some methodical flaws. The sampling method carries a risk of bias, as individuals with strong opinions may be more inclined to participate. The study sample consisted of only five participants, largely female, with amputations due to sepsis. The lived experiences with multiple amputations in this study may only reflect this specific subpopulation, and further research is needed to explore the experiences of men and those with amputations following major trauma. Thus, the results must be interpreted with caution. A potential limitation is that the analytical steps were primarily carried out by a single researcher, which may have influenced perspective and introduced bias. A more theoretically oriented design, where the lifeworld perspective served as the explicit foundation of the study (i.e., a deductive approach), could have further strengthened the theoretical consistency. Additionally, the study setting in Norway, with its public healthcare system and culture, may have influenced the participants’ expectations and life views, which may differ substantially from those in other countries and cultures. However, the themes identified in this study align with literature on other patient groups globally, and they may reflect universal aspects of living with a disability. Finally, it is important to note that the interviews were conducted during the COVID-19 pandemic, however the participants were asked to recall their experiences when discharged from specialized rehabilitation, which for all the participants were before the pandemic started. The pandemic might still have influenced the participants’ experiences and perceptions of their overall situation here and now, but to explore this further was outside the scope of this study.

## 8 Conclusion

This study highlights the profound challenges faced by individuals with multiple amputations after discharge from specialized rehabilitation. Participants described an existential struggle characterized by dependence, vulnerability, and a restricted lifeworld. The stark contrast between life with and without prosthetics underscores their essential role in promoting autonomy. Moreover, participants had high expectations of local rehabilitation services that were unmet, exposing structural barriers within community rehabilitation. To enhance individual rehabilitation outcomes, a holistic, lifelong, and patient-centered approach is recommended—one that integrates professional and social support, fosters independence, and recognizes each individual's unique needs and lived experience.

## Data Availability

The raw data supporting the conclusions of this article will be made available by the authors, without undue reservation.
